# Identification of Potential Anti-Neuroinflammatory Inhibitors from Antarctic Fungal Strain *Aspergillus* sp. SF-7402 via Regulating the NF-κB Signaling Pathway in Microglia

**DOI:** 10.3390/molecules27092851

**Published:** 2022-04-29

**Authors:** Thao Quyen Cao, Zhiming Liu, Linsha Dong, Hwan Lee, Wonmin Ko, Le Ba Vinh, Nguyen Quoc Tuan, Youn-Chul Kim, Jae Hak Sohn, Joung Han Yim, Dong-Sung Lee, Hyuncheol Oh

**Affiliations:** 1 Institute of Pharmaceutical Research and Development, College of Pharmacy, Wonkwang University, Iksan 54538, Korea; quyen.cao.thao@gmail.com (T.Q.C.); vinhrooney@gmail.com (L.B.V.); quoctuan301281@gmail.com (N.Q.T.); yckim@wku.ac.kr (Y.-C.K.); 2 Hanbang Cardio-Renal Syndrome Research Center, Wonkwang University, Iksan 54538, Korea; 3 College of Pharmacy, Chosun University, Dong-gu, Gwangju 61452, Korea; lzmqust@126.com (Z.L.); donglinsha011@163.com (L.D.); ghksdldi123@hanmail.net (H.L.); rabis815@naver.com (W.K.); 4 College of Medical and Life Sciences, Silla University, Busan 46958, Korea; jhsohn@silla.ac.kr; 5 Division of Polar Life Sciences, Korea Polar Research Institute, Incheon 21990, Korea; jhyim@kopri.re.kr

**Keywords:** Antarctica, fungi metabolites, neuroinflammation, BV2 cells, sterigmatocystin, NF-κB

## Abstract

Microglia play a significant role in immune defense and tissue repair in the central nervous system (CNS). Microglial activation and the resulting neuroinflammation play a key role in the pathogenesis of neurodegenerative disorders. Recently, inflammation reduction strategies in neurodegenerative diseases have attracted increasing attention. Herein, we discovered and evaluated the anti-neuroinflammatory potential of compounds from the Antarctic fungi strain *Aspergillus* sp. SF-7402 in lipopolysaccharide (LPS)-stimulated BV2 cells. Four metabolites were isolated from the fungi through chemical investigations, namely, 5-methoxysterigmatocystin (**1**), sterigmatocystin (**2**), aversin (**3**), and 6,8-*O*-dimethylversicolorin A (**4**). Their chemical structures were elucidated by extensive spectroscopic analysis and HR-ESI-MS, as well as by comparison with those reported in literature. Anti-neuroinflammatory effects of the isolated metabolites were evaluated by measuring the production of nitric oxide (NO), tumor necrosis factor (TNF)-α, and interleukin (IL)-6 in LPS-activated microglia at non-cytotoxic concentrations. Sterigmatocystins (**1** and **2**) displayed significant effects on NO production and mild effects on TNF-α and IL-6 expression inhibition. The molecular mechanisms underlying this activity were investigated using Western blot analysis. Sterigmatocystin treatment inhibited NO production via downregulation of inducible nitric oxide synthase (iNOS) expression in LPS-stimulated BV2 cells. Additionally, sterigmatocystins reduced nuclear translocation of NF-κB. These results suggest that sterigmatocystins present in the fungal strain *Aspergillus* sp. are promising candidates for the treatment of neuroinflammatory diseases.

## 1. Introduction

Neuroinflammation, an inflammatory response within the brain or spinal cord, is mediated by the production of cytokines, chemokines, reactive oxygen species, and secondary messengers. The role of these inflammatory mediators in the central nervous system (CNS) has been investigated in various neurodegenerative diseases such as Parkinson’s disease (PD), Huntington’s disease (HD), and Alzheimer’s disease (AD) [1,2]. Microglia play an active role in immune surveillance in the CNS by producing factors that influence surrounding astrocytes and neurons, particularly in response to pathogen invasion or tissue damage [3]. Rapid microglial activation reflects the response of the living tissue to injury to initiate wound healing and protect neurons from further damage. Hence, activated microglia are known to be neuroprotective [4,5]. Moreover, once activated, inflammatory microglial cells secrete increased levels of pro-inflammatory mediators, including nitric oxide (NO), inducible nitric oxide synthase (iNOS), cyclooxygenase (COX)-2, and pro-inflammatory cytokines such as tumor necrosis factor (TNF)-α and interleukin (IL)-6, which are associated with the activation of nuclear factor κ-light-chain-enhancer of activated B cells (NF-κB) pathways [6]. Various studies have shown that activation of NF-κB protects neurons against different injuries, such as oxidative stress, excitotoxicity, and amyloid β peptide toxicity. NF-κB has also been demonstrated as a main signal transducer that affects cellular permeability, endocytosis, and intracellular trafficking at the blood–brain barrier level. Thus, NF-κB plays a critical role in the regulation of neuroinflammation-associated disease pathogenesis [7].

To explore new bioactive natural products, researchers have invested a lot of effort to discover novel sources in different environments in recent years. Polar regions, which harbor diverse groups of fauna and microorganisms including bacteria, actinomycetes, and fungi, have drawn attention as rich sources of diverse secondary metabolites with significant anti-bacterial, anti-tumor, anti-virus, and anti-inflammatory effects [8,9]. Antarctica, which harbors a cold, dry climate with intense solar radiation, has nurtured a number of unique microbial resources that have been attracting increasing attention [10].

Our continued attempts to study bioactive metabolites isolated from Antarctic fungi led to the isolation of four secondary metabolites (**1**–**4**) from the fungal strain *Aspergillus* sp. SF-7402. The isolated metabolites were identified as 5-methoxysterigmatocystin (**1**), sterigmatocystin (**2**), aversin (**3**), and 6,8-*O*-dimethylversicolorin A (**4**). In our previous study, 5-methoxysterigmatocystin did not affect the expression of cobalt protoporphyrin (CoPP)-induced heme oxygenase (HO)-1 [6]. However, no clear study of the therapeutic mechanism of 5-methoxysterigmatocystin in LPS-stimulated BV2 cells has been performed to date. Moreover, the anti-neuroinflammatory effects of sterigmatocystin, aversin, and 6,8-*O*-dimethylversicolorin A have not yet been reported. Herein, we describe the isolation and structural characterization of isolated metabolites **1**–**4** and their anti-neuroinflammatory activity in LPS-stimulated BV2 microglial cells.

## 2. Materials and Methods

### 2.1. General Experimental Procedures

A Jasco P-2000 digital polarimeter (JASCO Corp., Tokyo, Japan) was used to record the optical rotations. HR-ESI-MS data were obtained using an electrospray ionization (ESI) quadrupole time-of-flight (Q-TOF) tandem mass spectrometry (MS/MS) system (AB SCRIEX Triple). Nuclear magnetic resonance (NMR) spectra (1D and 2D) were recorded in CDCl_3_ (δ_H_/δ_C_ = 7.26/77.16) using a JEOL JNM ECP-400 spectrometer (JEOL Ltd., Tokyo, Japan), and the chemical shifts were referenced relative to the residual solvent peaks. Heteronuclear multiple quantum coherence (HMQC) and heteronuclear multiple bond correlation (HMBC) experiments were optimized for ^1^*J*_CH_ = 140 Hz and ^n^*J*_CH_ = 8 Hz. Thin-layer chromatography (TLC) was performed on Kieselgel 60 F_254_ (1.05715; Merck, Darmstadt, Germany) or RP-18 F_254s_ (Merck, Darmstadt, Germany) plates. Spots were visualized by spraying plates with 10% aqueous sulfuric acid (H_2_SO_4_) solution, followed by heating. Column chromatography was performed on silica gel (Kieselgel 60, 70–230 mesh and 230–400 mesh, Merck, Darmstadt, Germany) and YMC octadecyl-functionalized silica gel (C_18_). High-performance liquid chromatography (HPLC) was performed on a preparative-C_18_ column (10 × 250 mm; 5 μm particle size) with a flow rate of 5 mL/min, and the compound was detected by ultraviolet (UV) absorption at 210 and 254 nm.

### 2.2. Fungal Material and Fermentation

The fungal strain SF-7402 was isolated from the lichen collected on King George Island (62°1406.15” S, 58°46′22.68” W), Antarctica, in January 2017. One gram of the sample was ground with a mortar and pestle and mixed with sterile seawater (10 mL). A portion (0.1 mL) of the sample was processed using the spread plate method in potato dextrose agar (PDA) medium containing seawater and incubated at 25 °C for 14 days. After sub-culturing the isolates several times, the final pure cultures were selected and stored at −70 °C. The fungal strain SF-7402 was identified using ribosomal RNA (rRNA) sequence analysis. A GenBank search with the ITS gene of SF-7402 (GenBank accession number MZ267531) indicated *Aspergillus jensenii* (NR_135444), *A. creber* (NR_135442), *A. versicolor* (NR_131277), and *A. protuberus* (NR_135353) as the closest matches showing sequence identities of 99.62%, 99.62%, 99.43%, and 99.43%, respectively. Therefore, the fungal strain SF-7402 was characterized as *Aspergillus* sp. A voucher specimen (SF-7402) was deposited at the Korea Polar Research Institute.

### 2.3. Extraction and Isolation of Metabolites

The fungal strain *Aspergillus* sp. SF-7402 was cultured in 20 Fernbach flasks, each containing 650 mL of PDA medium with 3% NaCl (*v*/*v*). The flasks were individually inoculated with 2 mL seed cultures of the fungal strain and incubated at 25 °C for 14 days. The fermented culture medium was combined and extracted with EtOAc (20 L). The combined EtOAc extracts were then filtered through filter papers and evaporated to dryness, resulting in a crude extract (4.8 g). The crude extract was fractionated by reversed-phase (RP) C_18_ flash column chromatography (30 × 4.5 cm), eluting with a stepwise gradient of 20%, 40%, 60%, 80%, and 100% (*v*/*v*) MeOH in H_2_O (500 mL each) to obtain five fractions, SF-7402-1 to SF-7402-5. Fraction SF-7402-5 (568.8 mg) was subjected to a Sephadex LH-20 column (33 × 3 cm) using CHCl_3_–MeOH (3:1, *v*/*v*) to obtain four fractions (SF-7402-5.1 to SF-7402-5.4). The fraction SF-7402-5.2 (521.6 mg) was chromatographed over a silica gel column (33 × 2 cm) and eluted with *n*-hexane–acetone (7:1, *v*/*v*) to yield ten sub-fractions (SF7402-5.2.1, SF7402-5.2.10). Finally, sub-fraction SF-7402-5.2.7 (153.9 mg) was separated using C_18_ prep HPLC (45–65% CH_3_CN in H_2_O, over 45 min) to obtain compounds **1** (25.3 mg, t_R_ = 30 min), **2** (15.8 mg, t_R_ = 34 min), **3** (9.5 mg, t_R_ = 39 min), and **4** (1.1 mg, t_R_ = 43 min).

#### 2.3.1. 5-Methoxysterigmatocystin (**1**)

Yellow needles; [α]_D_^20^ -378.5° (*c* = 0.2, CHCl_3_); ^1^H NMR (CDCl_3_, 400 MHz): 12.60 (s, O*H*-8), 7.16 (d, *J* = 8.8 Hz, H-6), 6.81 (d, *J* = 7.2 Hz, H-1′), 6.66 (d, *J* = 8.8 Hz, H-7), 6.48 (dd, *J* = 2.0, 2.8 Hz, H-4′), 6.41 (s, H-2), 5.49 (t, *J* = 2.8 Hz, H-3′), 4.83 (tdd, *J* = 7.2, 2.4, 0.4 Hz, H-2′), 3.98 (s, OC*H*_3_-1), 3.91 (s, OC*H*_3_-5); ^13^C NMR (CDCl_3_, 100 MHz): 181.3 (C-9), 164.7 (C-3), 163.4 (C-1), 155.3 (C-8), 154.0 (C-4a), 145.4 (C-4′), 144.8 (C-10a), 139.5 (C-5), 120.4 (C-6), 113.4 (C-1′), 109.6 (C-7, C-8a), 106.9 (C-4), 106.1 (C-9a), 102.7 (C-3′), 90.7 (C-2), 57.8 (O*C*H_3_-5), 56.9 (O*C*H_3_-1), 48.2 (C-2′). HR-ESI-MS *m/z*: 355.0814 [M + H]^+^ (calculated for [C_19_H_15_O_7_]^+^, 355.0812).

#### 2.3.2. Sterigmatocystin (**2**)

Yellow needles; [α]_D_^20^ -393.8° (*c* = 0.12, CHCl_3_); ^1^H NMR (CDCl_3_, 400 MHz): 13.22 (s, O*H*-8), 7.49 (d, *J* = 8.0 Hz, H-6), 6.82 (d, *J* = 6.8 Hz, H-1′), 6.82 (dd, *J* = 8.4, 1.2 Hz, H-5), 6.75 (dd, *J* = 8.4, 0.8 Hz, H-7), 6.50 (dd, *J* = 2.0, 2.4 Hz, H-4′), 6.43 (s, H-2), 5.44 (t, *J* = 2.8 Hz, H-3′), 4.81 (dt, *J* = 7.2, 2.0 Hz, H-2′), 3.99 (s, OC*H*_3_-1); ^13^C NMR (CDCl_3_, 100 MHz): 181.5 (C-9), 164.7 (C-3), 163.4 (C-1), 162.5 (C-8), 155.1 (C-10a), 154.2 (C-4a), 145.5 (C-4′), 135.8 (C-6), 113.4 (C-1′), 111.4 (C-7), 109.1 (C-8a), 106.6 (C-9a), 106.1 (C-4), 106.0 (C-5), 102.6 (C-3′), 90.6 (C-2), 56.9 (O*C*H_3_-1), 48.2 (C-2′). HR-ESI-MS *m/z*: 325.0693 [M + H]^+^ (calculated for [C_18_H_13_O_6_]^+^, 325.0707).

#### 2.3.3. Aversin (**3**)

Yellow solid; [α]_D_^20^ -117.2° (*c* = 0.12, CHCl_3_); ^1^H NMR (CDCl_3_, 400 MHz): 13.49 (d, *J* = 0.4 Hz, O*H*-1), 7.43 (d, *J* = 2.4 Hz, H-5), 7.20 (s, H-4), 6.77 (d, *J* = 2.4 Hz, H-7), 6.45 (d, *J* = 6.0 Hz, H-1′), 4.14 (dt, *J* = 8.8, 6.8 Hz, H-2′, H_β_-4′), 4.02 (s, OC*H*_3_-8), 3.97 (s, OC*H*_3_-6), 3.65 (ddd, *J* = 13.1, 8.8, 4.8 Hz, H_α_-4′), 2.36 (dd, *J* = 12.4, 4.8 Hz, H_α_-3′), 2.25 (m, H_β_-3′); ^13^C NMR (CDCl_3_, 100 MHz): 187.3 (C-9), 182.6 (C-10), 165.2 (C-3), 165.1 (C-6), 163.0 (C-8), 160.4 (C-1), 137.6 (C-10a), 135.0 (C-4a), 120.2 (C-2), 115.1 (C-8a), 113.1 (C-1′), 112.7 (C-9a), 105.0 (C-7), 104.2 (C-5), 101.3 (C-4), 67.9 (C-4′), 56.8 (O*C*H_3_-8), 56.2 (O*C*H_3_-6), 44.5 (C-2′), 30.9 (C-3′). HR-ESI-MS *m/z*: 369.0949 [M + H]^+^ (calculated for [C_20_H_17_O_7_]^+^, 369.0969).

#### 2.3.4. 6,8-*O*-dimethylversicolorin A (**4**)

Yellow powder; [α]_D_^20^ -93.2° (*c* = 0.07, CHCl_3_); ^1^H NMR (CDCl_3_, 400 MHz): 13.41 (s, O*H*-1), 7.45 (d, *J* = 2.4 Hz, H-5), 7.30 (d, *J* = 0.8 Hz, H-4), 6.80 (d, *J* = 7.6 Hz, H-1′), 6.79 (d, *J* = 2.4 Hz, H-7), 6.48 (dd, *J* = 2.4, 2.8 Hz, H-4′), 5.46 (t, *J* = 2.4 Hz, H-3′), 4.73 (dtd, *J* = 7.6, 2.4, 0.8 Hz, H-2′), 4.03 (s, OC*H*_3_-8), 3.98 (s, OC*H*_3_-6); ^13^C NMR (CDCl_3_, 100 MHz): 187.3 (C-9), 182.6 (C-10), 165.3 (C-6), 163.6 (C-3), 163.1 (C-8), 159.9 (C-1), 145.5 (C-4′), 137.6 (C-10a), 134.8 (C-4a), 121.0 (C-2), 113.2 (C-8a, C-1′), 112.9 (C-9a), 105.0 (C-7), 104.3 (C-5), 102.1 (C-3′), 102.0 (C-4), 56.8 (O*C*H_3_-8), 56.2 (O*C*H_3_-6), 48.5 (C-2′). HR-ESI-MS *m/z*: 389.0609 [M + Na]^+^ (calculated for [C_20_H_14_NaO_7_]^+^, 389.0632).

### 2.4. Cell Culture and Reagents

Tissue culture reagents, such as Roswell Park Memorial Institute 1640 (RPMI1640) and fetal bovine serum, were purchased from Gibco BRL Co. (Grand Island, NY, USA). All chemicals were obtained from Sigma-Aldrich Chemical Co. (St. Louis, MO, USA). Primary antibodies, including anti-iNOS, anti-COX-2, anti-pIκBα, anti-β-actin, anti-p65, and anti-PCNA, were purchased from Santa Cruz Biotechnology (Santa Cruz, CA, USA); all the primary antibodies were rabbit source. Anti-rabbit secondary antibodies were purchased from Millipore (Billerica, MA, USA). Enzyme-linked immunosorbent assay (ELISA) kits for IL-6 and TNF-α were purchased from R&D Systems, Inc. (Minneapolis, MN, USA).

### 2.5. Cell Viability Assay

The BV2 cells were incubated at 5 × 10^5^ cells/mL in RPMI1640 containing 1% antibiotics (penicillin-streptomycin) and 10% heat-inactivated FBS at 37 °C in a humidified 5% CO_2_ and 95% air atmosphere. Mitochondrial reductase reduces the tetrazolium salt 3-[4,5-dimethylthiazol-2-yl]-2,5-diphenyltetrazolium bromide (MTT) to formazan crystals. Using this, the effect of compounds **1**, **2**, **3**, and **4** on cell viability was measured. To measure cell viability, 5 mg/mL MTT was treated with each cell suspension (1 × 10^5^ cells/mL) to form formazan for 4 h. Formazan formed was dissolved in DMSO, and absorbance was measured at 540 nm [11,12].

### 2.6. Measurement of Nitrite Oxide (NO) Generation

The concentration of nitrite in the conditioned media was determined using a method based on the Griess reaction [13]. Nitrite levels were measured at 570 nm after the cell culture solution and Griess reagent were mixed at the same volume and allowed to react.

### 2.7. Assays for IL-6 and TNF-α Production

The culture medium was collected to determine the levels of IL-6 and TNF-α present in each sample using a commercially available kit (BioLegend, San Diego, CA, USA). The assay was performed according to the manufacturer’s instructions. Briefly, BV2 cells were seeded in 24-well culture plates at a density of 2 × 10^5^ cells/well. After incubation, the supernatant was collected and used in the cytokine ELISA kits to measure the concentrations of IL-6 and TNF-α.

### 2.8. Determination of Prostaglandin E2 (PGE_2_) Levels

The culture medium was collected to determine the level of PGE_2_ present in each sample. The assay was performed according to the manufacturer’s instructions. Briefly, BV2 cells were seeded in 24-well culture plates at a density of 2 × 10^5^ cells/well. After incubation, supernatants were collected. Then, PGE_2_ levels were measured using a specific ELISA kit from R&D Systems, Inc. (Minneapolis, MN, USA).

### 2.9. Analysis of iNOS and COX-2 Protein Expression

The pelleted BV2 cells were washed with phosphate-buffered saline and lysed in RIPA buffer. Equal amounts of proteins were quantified by the protein assay dye reagent concentrate obtained from Bio-Rad Laboratories (#5000006; Hercules, CA, USA), mixed in the sample loading buffer, and separated by SDS-PAGE. The separated proteins were then transferred onto nitrocellulose membranes. Non-specific binding to the membrane was blocked by incubation in a solution of skim milk. The membrane was incubated with primary antibodies at 4 °C overnight and then reacted with a horseradish peroxidase-conjugated secondary antibody (Millipore).

### 2.10. Analysis of NF-κB (p65) Expressions

To study the localization of NF-κB, BV2 cells were seeded in 24-well culture plates at a density of 40 × 10^5^ cells/well and were treated with concentrations of compounds **1** and **2** for 3 h before LPS stimulation (0.5 μg/mL) for 1 h. A nuclear extraction kit (Cayman, Ann Arbor, MI, USA) was used to separate cytosolic and nuclear fractions. Each extracted fraction was lysed according to the protocol provided by the manufacturer. Then, the expression of NF-κB (p65) pathway was determined by Western blot analysis. Equal amounts of proteins were quantified by the protein assay dye reagent concentrate obtained from Bio-Rad Laboratories (#5000006; Hercules, CA, USA), mixed in the sample loading buffer, and separated by SDS-PAGE. The separated proteins were then transferred onto nitrocellulose membranes. Non-specific binding to the membrane was blocked by incubation in a solution of skim milk., the membrane of cytosolic fraction sample was incubated with pIκBα antibody at 4 °C overnight, while the membrane of nuclear fraction sample was incubated with p65 antibody and then reacted with a horseradish peroxidase-conjugated secondary antibody (Millipore).

### 2.11. Statistical Analysis

Data are presented as mean ± standard deviation of three independent experiments. One-way analysis of variance, followed by Tukey’s multiple comparison test, was used to compare the three groups. Statistical analyses were performed using GraphPad Prism software, version 5.01 (GraphPad Software Inc., San Diego, CA, USA).

## 3. Results

### 3.1. Structure Elucidation and Effects of Isolated Metabolites on BV2 Cells Viability

**Figure 1:**
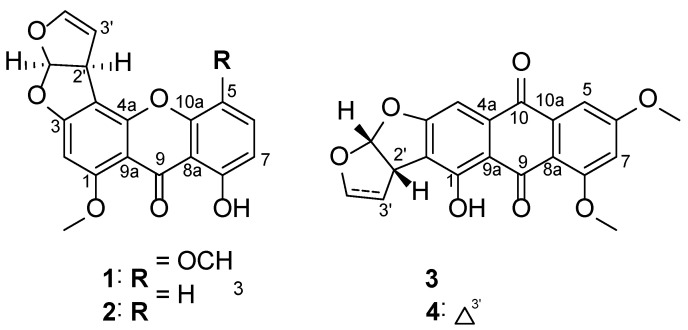
Structure of isolated metabolites (**1**–**4**).

To identify the bioactive metabolites responsible for the anti-neuroinflammatory effects, efficient chromatographic separation techniques were adapted for the isolation of four compounds (**1**–**4**) from the fungal strain *Aspergillus* sp. SF-7402 (Figure 1), which were characterized as 5-methoxysterigmatocystin (**1**), sterigmatocystin (**2**), aversin (**3**), and 6,8-*O*-dimethylversicolorin A (**4**), by comparison of their spectral data with values reported in literature [14].

**Figure 2:**
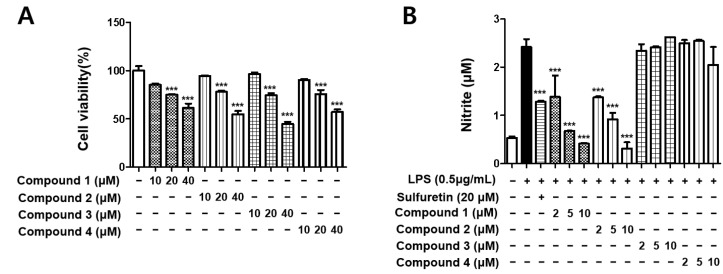
The effects of compounds on cell viability (**A**) and nitrite content (**B**). BV2 cells were incubated for 24 h with various concentrations of compounds **1**–**4**. Cell viability was determined using the 3-(4,5-dimethylthiazol-2-yl)-2,5-diphenyltetrazolium bromide assay. BV2 cells were pretreated for 3 h with indicated concentrations of compounds and stimulated for 24 h with lipopolysaccharide (LPS) (0.5 μg/mL). Bars represent means ± standard deviation of three independent experiments. *** *p* < 0.001 compared with LPS-treated group.

To avoid the toxic effects of the isolated metabolites, we determined the viability of BV2 cells following treatment for 24 h at concentrations ranging from 10 to 40 μM using the MTT assay. As shown in Figure 2A, all four compounds (**1**–**4**) demonstrated cell toxicity in BV2 cells at concentrations of 20 and 40 μM. Thus, further experiments were conducted using each compound in the non-toxic concentration range (2–10 μM). NO, a neurotransmitter and second messenger molecule, mediates various physiological and pathological processes in many organ systems, including the brain [15]. To examine the inhibitory effects of isolated metabolites (**1**–**4**) on the LPS-induced production of NO in BV2 cells, the cell culture medium was harvested, and nitrite concentrations were measured using the Griess reaction [13,14]. The results showed that two xanthones, 5-methoxysterigmatocystin (**1**) and sterigmatocystin (**2**), significantly inhibited NO production in LPS-induced BV2 cells, while two anthraquinones, aversin (**3**) and 6,8-*O*-dimethylversicolorin A (**4**), did not inhibit NO production (Figure 2B).

### 3.2. Sterigmatocystins Inhibited LPS-induced Expression of iNOS and COX-2 Proteins in BV2 Cells

**Figure 3:**
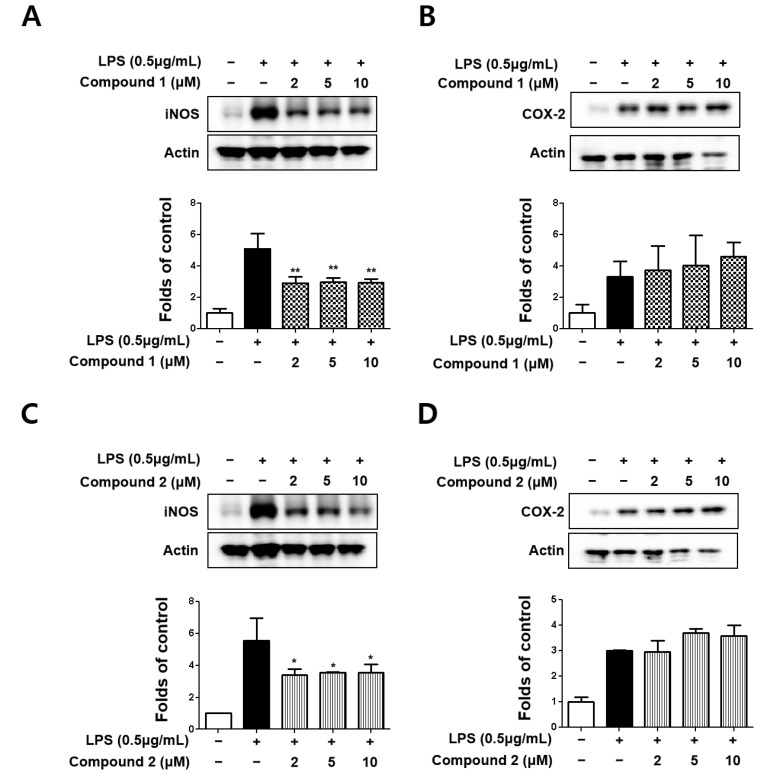
Protein expression levels of inducible nitric oxide synthase (iNOS) (**A**,**C**) and cyclooxygenase-2 (COX-2) (**B**,**D**) in lipopolysaccharide (LPS)-stimulated BV2 cells. Cells were pretreated for 3 h with indicated concentrations of compounds **1** and **2** and stimulated for 24 h with LPS (0.5 μg/mL). Representative blots from three independent experiments are shown. Immunoblots were quantified using ImageJ software. Band intensities were normalized to β-actin. * *p* < 0.05, ** *p* < 0.01 compared with LPS-treated group.

Subsequently, the effects of sterigmatocystins (**1** and **2**) on LPS-induced expression of iNOS and COX-2 proteins in BV2 cells were investigated by Western blot analysis. Both the sterigmatocystins (**1** and **2**) were found to inhibit LPS-induced iNOS expression but did not affect COX-2 expression (Figure 3). The results of the present study suggest that sterigmatocystins with a xanthone skeleton are promising compounds for selective inflammatory inhibition in LPS-stimulated BV2 cells.

### 3.3. Sterigmatocystins Inhibited the LPS-induced Production of Pro-inflammatory Cytokines in BV2 Cells

**Figure 4:**
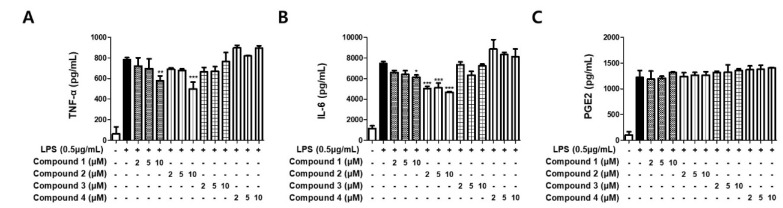
Effects of the isolated compounds on TNF-α (**A**), IL-6 (**B**), and PGE_2_ (**C**) in LPS-stimulated BV2 cells. Cells were pretreated for 3 h with indicated concentrations of compounds 1–4 and stimulated for 24 h with LPS (0.5 μg/mL). Bars represent means ± standard deviation of three independent experiments. * *p* < 0.05, ** *p* < 0.01, *** *p* < 0.001 compared with LPS-treated group.

To evaluate the inhibitory effects on the expression of pro-inflammatory cytokines TNF-α, IL-6, and PGE_2_, the isolated metabolites (**1**–**4**) were used for the treatment of LPS-stimulated BV2 cells. The experiments were performed using ELISA kits. As shown in Figure 4, only sterigmatocystins (**1** and **2**) displayed mild potent inhibitory effects on both TNF-α and IL-6 expression, whereas the other isolated compounds did not inhibit the expression. Compound **1** decreased TNF-α and IL-6 expression in the cells by approximately 25% and 20%, respectively, at a concentration of 10 μM. Compound **2** exhibited over 30% potent inhibition of TNF-α expression in activated BV2 cells at a concentration of 10 μM, and over 30% downregulation of IL-6 expression at all tested concentrations (2, 5, and 10 μM). However, pretreatment of sterigmatocystins (**1** and **2**) was not able to abrogate PGE_2_ production in LPS-stimulated BV2 cells, which reflect the same effects of sterigmatocystins (**1** and **2**) on iNOS and COX-2 expression. These data demonstrate the roles of sterigmatocystins (**1** and **2**) in the inhibition of pro-inflammatory cytokine expression.

### 3.4. Sterigmatocystins Inhibited the LPS-induced Activation of NF-κB Pathway in BV2 Cells

**Figure 5:**
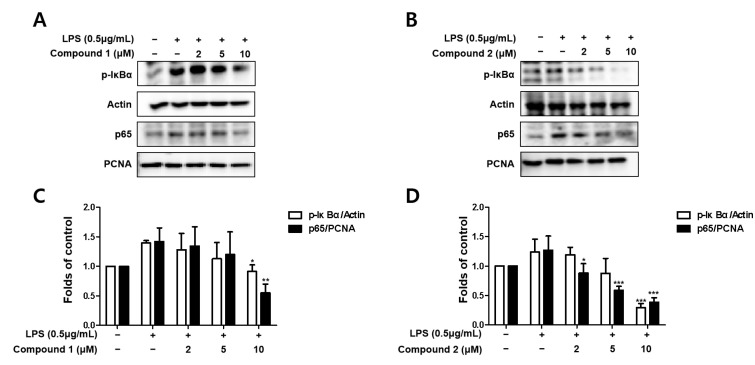
Effects of the isolated compounds on the NF-κB (p65) pathway in BV2 cells (**A**–**D**). Cells were pretreated with indicated concentrations of compounds 1 and 2 for 3 h and stimulated with lipopolysaccharide (LPS, 1 μg/mL) for 1 h. Representative blots from three independent experiments are shown. Immunoblots were quantified using ImageJ software. p-IκBα intensities were normalized to β-actin. P65 intensities were normalized to PCNA. * *p* < 0.05, ** *p* < 0.01, *** *p* < 0.001 compared with LPS-treated group.

Since the abnormal regulation of NO, TNF-α, and IL-6 in the activated microglia are related to the activation of the NF-κB (p65) pathway, we further evaluated whether sterigmatocystins (1 and 2) upregulated NF-κB activation. BV2 cells were pretreated with the indicated concentrations of compounds 1 and 2 for 3 h and then stimulated with LPS (1 μg/mL) for 1 h. The cytosolic and nuclear fractions were extracted from the cell lysate, and protein levels of p65 increased in the nuclear fraction of the LPS-treated group. Pretreatment with compounds 1 and 2 decreased p65 expression in a concentration-dependent manner (Figure 5). The phosphorylation of IκB-α in the cytosolic fraction was also increased by LPS stimulation; however, compounds 1 and 2 inhibited this response in a concentration-dependent manner (Figure 5). These results suggest that sterigmatocystins exhibit anti-neuroinflammatory effects by suppressing NF-κB pathway activation. 

## 4. Discussion

Neuroinflammation is initially a protective mechanism, where inflammatory mediators work to restore damaged neuronal and glial cells during acute neuroinflammation [16,17,18,19,20]. However, chronic neuroinflammation tends to cause more neuronal damage and eventual degradation [21,22,23,24,25,26,27,28,29]. Finding therapeutic medicines for neuroinflammation could potentially decrease the progression of neurodegenerative diseases.

Antarctic microbes, especially fungi, have been proven to be a rich source of novel secondary metabolites [22]. The metabolites derived from Antarctic fungi have been categorized into polyketides, peptides, alkaloids, and terpenoids, which show potent anti-inflammatory, anticancer, antimicrobial, and protein tyrosine phosphatase 1 B (PTP1B) inhibitory activities [22,23]. In this study, BV2 cells were used to explore the anti-neuroinflammatory effects of the four metabolites isolated from *Aspergillus* sp. SF-7402, which may provide a new strategy for the treatment of neuroinflammation.

Nitric oxide is enzymatically formed from arginine by the three nitric oxide synthase (NOS) isoforms. Inducible NOS (iNOS), an isoform of NOS family, can be induced by LPS and various cytokines [24]. iNOS is expressed in many cell types, including microglial cells. In the healthy brain, microglial cells do not express iNOS; nevertheless, under ischemic, traumatic, neurotoxic, or inflammatory damage, they become activated to produce iNOS and release a large amount of NO [25]. Thereafter, the cell membrane structure can be damaged, affecting DNA transcription and protein synthesis, and directly damage neurons. NO causes neuronal degeneration by changing the intracellular Fe^2+^ concentration. Hence, knockdown of iNOS in microglial cells inhibits the production of NO, thereby reducing CNS degeneration [26]. The results showed that two xanthones, 5-methoxysterigmatocystin and sterigmatocystin, significantly inhibited NO production (Figure 2B). Subsequently, the effects of sterigmatocystins (**1** and **2**) on LPS-induced expression of iNOS and COX-2 proteins in BV2 cells were investigated by Western blot analysis. Both sterigmatocystins (**1** and **2**) inhibited LPS-induced iNOS expression but did not affect COX-2 expression (Figure 3). Although the signaling pathways for expression of COX-2 and iNOS are complex, these differential effects may be due to the degree of dependency of iNOS and COX-2 promoters on the various transcription factors. iNOS promoter contains several cis-acting elements such as NF-κB, AP-1, C/EBPβ, and Stat, while COX-2 promoter has NF-κB, C/EBPβ, and CRE cis-acting elements [27,28]. The activity of these promoters may vary according to the cell type and the stimulus applied.

In supporting this notion, the expression of other pro-inflammatory cytokines such as TNF-α and IL-6, which was known to be critically dependent on the activation of NF-κB, was significantly inhibited by compounds **1** and **2** (Figure 4A,B). Among the immune mediators released from activated microglial cells, NO and pro-inflammatory cytokines, including TNF-α and IL-6, are important mediators in the development of neuroinflammation. Abundant evidence has revealed that cytokines such as TNF-α and IL-6 can be indicators of microglial activation [29]. TNF-α released from brain cells is the center of neuroinflammatory responses under pathological conditions [30]. IL-6 is one of the factors that cause acute neuroinflammatory reactions to inflammatory injury [31]. PGE_2_, the product of COX-2 enzyme, is also regulated by NF-κB and plays an important role in neuroinflammation [32]; however, compounds **1** and **2** were not able to inhibit PGE_2_ production (Figure 4C), which reflects the same effects of compounds **1** and **2** on COX-2 expression. These results suggest that the regulative effect of compounds **1** and **2** shows less dependency on COX-2 promoter compared to iNOS promoter in LPS-stimulated BV2 cells.

Chronic neuroinflammation is primarily controlled by microglial cells and resident immune cells in the brain. Chronic neuroinflammation is known to be a major reason for the loss of neurons in PD and forms the basis of neurodegeneration [33]. NF-κB is a member of the Rel family of transcription factors. RelA (p65) is one of the five members of the Rel family. The post-transcriptional modifications of p65 play a crucial role in the onset of neurodegenerative processes activated by ischemic insults and glutamate or β-amyloid toxicity [34]. IκB proteins, as essential regulators of nuclear import, can interact with nuclear localization signals to induce an alpha helical conformation. The most important IκB protein is thought to be IκB-α because it only interacts with the nuclear localization signal of p65 [35]. Pretreatment with compounds **1** and **2** decreased p65 expression in a concentration-dependent manner (Figure 5). The phosphorylation of IκB-α in the cytosolic fraction was also increased by LPS stimulation; however, compounds **1** and **2** inhibited this response in a concentration-dependent manner (Figure 5). These results suggest that sterigmatocystins further confirm the anti-neuroinflammatory effects by suppressing NF-κB pathway activation. 

Sterigmatocystin is a xanthone containing xanthone and bisfuran groups [36]. Sterigmatocystin derivatives have been reported to possess a large number of biological activities, including anti-tumor, anti-inflammatory, antiviral [37], and antibacterial activities [38]. Among the isolated sterigmatocystins, compound **2**, which differs from compound **1** by the absence of the methoxy group at position C-5, demonstrated a greater effect on the expression of p65 protein and phosphorylation of IκB-α at the indicated concentrations in LPS-stimulated BV2 cells (Figure 5B). This point indicates the key role of the methoxy group in the aromatic rings of sterigmatocystins in its anti-neuroinflammatory activity.

## 5. Conclusions

Herein, we identified two xanthones, sterigmatocystins (1, 2), and two anthraquinones (3, 4) present in the Antarctic fungal strain *Aspergillus* sp. SF-7402. We demonstrated that sterigmatocystins exhibited remarkable anti-neuroinflammatory effects by inhibiting the production of NO, TNF-α, and IL-6, and the expression of iNOS protein. These effects were mediated via activation of the NF-κB pathway in LPS-challenged BV2 microglial cells. Consequently, these findings provide promising substrates for the development of therapeutic agents for the treatment of neurodegenerative diseases.

## Data Availability

The data presented in this study are available in this article. Other data that support the findings of this study are available upon request from the corresponding author.

## Sample Availability

Samples of the compounds are not available from the authors.
